# The effects of lateral meniscus posterior root tears or repair with anterior cruciate ligament reconstruction on the pressure of the patellofemoral joint: A biomechanical evaluation

**DOI:** 10.12669/pjms.39.2.6692

**Published:** 2023

**Authors:** Qian Zhang, Jiangtao Dong, Xiaozuo Zheng, Kai Kang, Shijun Gao

**Affiliations:** 1 Qian Zhang, Department of Orthopedics surgery, The Third Hospital of Hebei Medical University, Shijiazhuang 050051, Hebei, P. R. China; 2 Jiangtao Dong, Department of Orthopedics surgery, The Third Hospital of Hebei Medical University, Shijiazhuang 050051, Hebei, P. R. China; 3 Xiaozuo Zheng, Department of Orthopedics surgery, The Third Hospital of Hebei Medical University, Shijiazhuang 050051, Hebei, P. R. China; 4 Kai Kang, Department of Orthopedics surgery, The Third Hospital of Hebei Medical University, Shijiazhuang 050051, Hebei, P. R. China; 5 Shijun Gao, Department of Orthopedics surgery, The Third Hospital of Hebei Medical University, Shijiazhuang 050051, Hebei, P. R. China

**Keywords:** Patellofemoral contact pressure, Lateral meniscus, Posterior root tear, ACL reconstruction

## Abstract

**Objective:**

The aim of our study was to evaluate the effect of tear and repair of the lateral meniscal posterior root (LMPR) on the patellofemoral contact pressure of the knee after anterior cruciate ligament (ACL) reconstructed.

**Methods:**

This was a descriptive study. Six fresh-frozen cadaveric knees collected by The Third Hospital of Hebei Medical University from January 2019 and January 2022 were placed on a customized testing rig. Patellofemoral contact pressures were measured at 30°, 60°, and 90° of flexion using pressure-sensitive film inserted between the patella and trochlea. The following knee states were tested: ACL reconstruction and intact lateral meniscus, ACL reconstruction, and LMPR tear, and ACL reconstruction and LMPR repair. Pressure measurements were recorded for each state.

**Result:**

In the ACL-reconstructed knee, a tear of the LMPR increased patellofemoral contact pressure at 30° of knee flexion. The repair of the posterior root by transosseous pull-out suture reduced the patellofemoral contact pressure as the status of intact lateral meniscal posterior root at 30° of knee flexion. There was no statistical difference between ACL reconstruction with the intact meniscal root and with the meniscal root tear and with the meniscal root repair at 60° and 90° of knee flexion.

**Conclusion:**

The posterior root tear and repair of the lateral meniscus could have an influence on patellofemoral contact stress of the knee after ACL reconstruction at 30° of knee flexion.

## INTRODUCTION

A meniscal root tear is described as a meniscal tear in the scope of less than one cm from the insertion, or abruption of the meniscal attachment.^1^ The meniscus root tear could inhibit axial loads from changing into transversal stresses and accelerate cartilage degeneration.^2^ As a concomitant injury of an anterior cruciate ligament (ACL) tear, the lateral meniscal posterior root tear (LMPR) is frequently found with incidence from 8% to 14%.^3^,^4^ Presently, there is no consensus on the LMPR tear at the time of ACL reconstruction.^5^ The recent trend has been to repair meniscal tears to restore joint contact biomechanics as much as possible to the normal state because several studies have shown repairs of the lateral meniscal tears significantly decreased the lateral compartment contact pressure.^6^

Most of the published literature regarding lateral meniscal posterior root tears concurrent ACL reconstruction has focused on the change of kinematics, clinical results, and on the cartilage degeneration of lateral compartment, not patellofemoral osteoarthritis.^7^-^9^ No study has evaluated the effect of lateral meniscal posterior root tear or repair on patellofemoral contact pressures at the time of ACL reconstruction, which is usually done in clinical practice. The purpose of our study was to evaluate whether there is a difference in the patellofemoral contact pressures among LMPR intact, tears, repairs after ACL reconstruction at simulated knee flexion. We hypothesized that the tear of the lateral meniscus posterior root would improve patellofemoral contact pressures during flexion state, while the repair of it would decrease the contact pressure reach to the uninjured state.

## METHODS

This was a descriptive study. Six fresh-frozen cadaveric human knee specimens (mean age 60 years, range 53 to 72) collected by The Third Hospital of Hebei Medical University from January 2019 and January 2022 fixed on the test rig were tested using a pressure sensor (Tekscan). Each specimen was thawed at room temperature overnight before testing. The femur and tibia were cut approximately 20 cm from the joint line, and bone ends were fixed by clamps on the rig. Soft tissues around the knee joint (including ligaments, muscles, and skin) were left intact. Each specimen was preconditioned by manually flexing the knee 10 times between full extension and full flexion before testing. The fix rig was used to simulate the flexion angles of 30°, 60°, 90° under simulated muscle loads, Using hanging weights, the quadriceps and iliotibial band were loaded to 175 N and 30 N of tension, respectively, and these were applied following the directions and cross-sectional areas of the muscles, as described in previous studies.^10^ The study was approved by the Institutional Ethics Committee of The Third Hospital of Hebei Medical University (No.:2020020; Date: March 20, 2020), and written informed consent was obtained from all participants.

### Surgical technique Anatomic:

**Fig.1:**
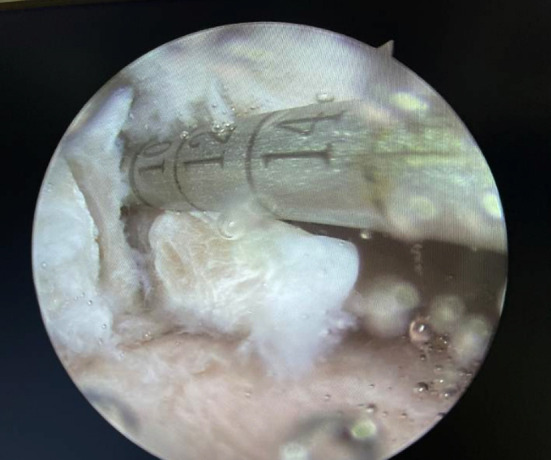
The lateral posterior root was cut.

Single-bundle arthroscopic ACL reconstruction was accomplished on all the specimens by the same surgeon, technique, and operative setting. The hamstring tendon of the cadaveric specimen was excised and prepared into 7-mm diameter auto grafts for ACL reconstructions. Application of a No. 11 surgical blade and a motorized shave to remove the ACL. The femoral tunnel was prepared via the anteromedial portal by applying a 7 mm femoral reamer at the center of the ACL insertion. Then, drilling of the tibial tunnel was done at the footprint area of the ACL tibial attachment using a 7 mm reamer and a 55° tibial drill guide system (Smith & Nephew Endoscopy). The lateral femur of the graft was fixed with an extracortical button, and fixation of the tibial segment of the graft was with an interface screw at 30° of knee flexion. The LMPR was cut by a No.11 blade under arthroscopy for demonstrating posterior root injure and was confirmed mobility by a probe (Fig.1).

**Fig.2:**
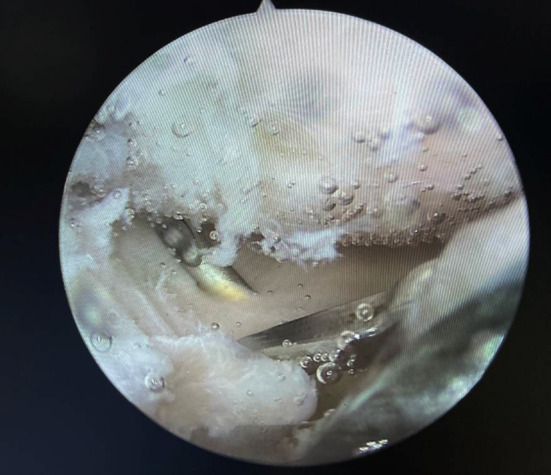
The posterior root of the meniscus was crossed by the needle.

**Fig.3:**
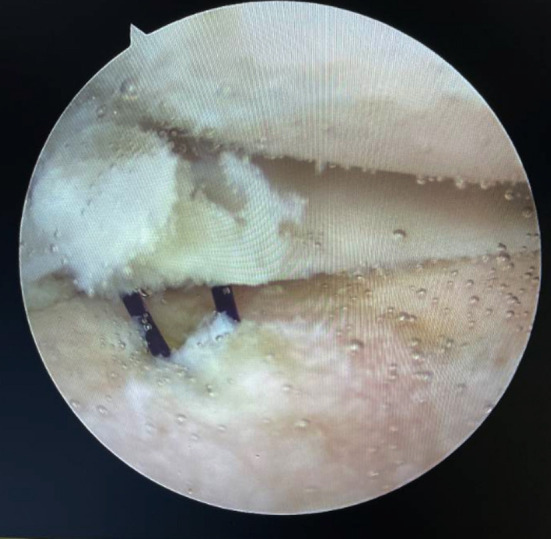
A suture was pulled out the prepared tunnel.

For the posterior root repaired, the 4.5 mm tibial tunnel was drilled in the center of the footprint of the meniscus root using a guide (Smith & Nephew Endoscopy) to locate the tunnel accurately. The prepared tunnel was covered by the posterior root of the meniscus with a grasper and then the posterior root of the meniscus was crossed by the needle with the suture retriever’s loop through the prepared tunnel (Fig.2). After the passage of the needle, the suture’s loop as a retriever was pulled out through the lateral portal with a grasper for guiding a suture to pass the posterior root and the prepared tunnel (Fig.3). By using the same method, the other end of the suture is pierced through the posterior root, meanwhile, a knotless suture anchor can be used to fasten the pulled-out suture.

### Contact pressure measurements:

Contact pressures were measured with a pressure sensor (TekScan, Boston). The pressure sensor was placed in the patellofemoral compartment via a lateral incision of the patellar. The sensors were calibrated before each test. At 30°, 60°, and 90°of knee flexion, Patellofemoral contact pressures were measured at three different knee states, in the following order: (1) ACL reconstruction with intact lateral meniscus, (2) ACL reconstruction with LMPR tear, and (3) ACL reconstruction with LMPR repair.

### Statistical analysis:

Different states of the posterior root of the lateral meniscus after ACL reconstruction were independent variables and patellofemoral joint pressure data were dependent variables. The data was verified to be normally distributed. The two-tailed Student’s t-test and paired t-test were used to compare whether there was a statistical difference in patellofemoral joint pressure between the two groups in the three states of intact, torn, and repaired posterior root of the lateral meniscus after ACL reconstruction. Statistical significance was set at p<0.05.

## RESULTS

**Fig.4:**
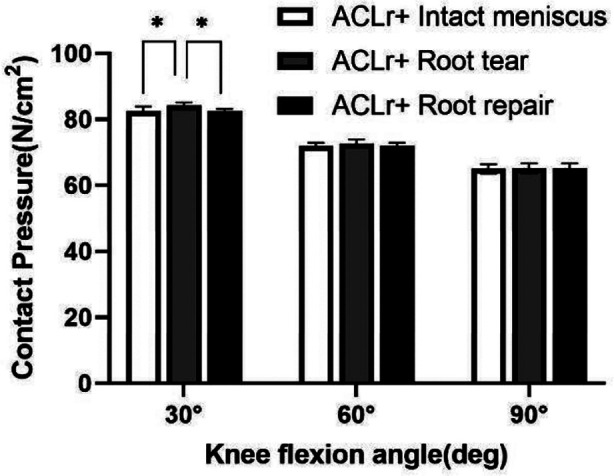
Patellofemoral contact pressure of knees in different states at different flexion angle. Values are presented as mean ± SD. *p<0.05. ACLr, anterior cruciate ligament reconstruction.

After the lateral menisci root tear, the patellofemoral contact pressures of the ACL-reconstructed knee increased significantly in comparison with the knee after ACL reconstruction with an intact meniscus at 30°(p=0.002) of knee flexion. After the root repair, the contact pressures of the patellofemoral joint decreased significantly versus ACL reconstruction with the root tear at 30° (p=0.001) of knee flexion. The repair of the posterior root returned the patellofemoral contact pressures to levels not statistically different from those of the ACL-reconstructed knee with an intact meniscus at 30° (p=0.818) of knee flexion (Fig.4). While there was no statistical difference between ACL reconstruction with the intact meniscal root and with the meniscal root tear and with the meniscal root repair at 60° and 90° of knee flexion.

## DISCUSSION

This study aimed to assess the patellofemoral contact pressure in the state of intact, torn, and repaired posterior root of the lateral meniscus after ACL reconstruction at 30° 60°and 90°of knee flexion. Our findings showed that a tear in the posterior root of the lateral meniscus significantly increased patellofemoral contact pressures compared to an intact or repaired posterior root of the lateral meniscus after ACL reconstruction at 30° of knee flexion. Moreover, posterior root repair of the lateral meniscus can significantly reduce patellofemoral joint pressure of the knee with ACL reconstruction at 30° of knee flexion. Furthermore, the results were in line with our hypothesis that in patients with ACL injury combined with posterior root injury of the lateral meniscus, the tear, and repair of the posterior root of the lateral meniscus affects the patellofemoral joint pressure at specific angles after ACL reconstruction.

Previous literature had reported that posterior root injuries to the lateral meniscus are a common comorbid injury in ACL injuries.^3^,^4^ There was still no consensus on the treatment of posterior root injuries of the lateral meniscus after ACL reconstruction. Satisfactory results had been reported for posterior meniscus root tears both left in situ and repaired.^11^-^13^ For this comorbid injury, the effect of different treatment modalities on the posterior root of the lateral meniscus on the patellofemoral joint was unknown. Our findings indicated that after ACL reconstruction, a tear of the posterior root of the lateral meniscus increased pressure on the patellofemoral joint at 30 degrees of flexion and that repair decreased patellofemoral joint pressure. The reasons resulting these outcomes are not known.

It was considered as a reason that the abnormal rotation of the tibia as a result of the tear of the LMPR in the early stages of knee flexion. Alternatively, increased knee valgus may also embolden the outcomes. It had been reported in some studies^14^ that a tear of the posterior root of the lateral meniscus could enhance knee valgus and induce an abnormal patellofemoral trajectory. However, patellofemoral joint pressure did not differ significantly between the posterior meniscus root repair and the posterior meniscus root intact for the knee with ACL reconstruction. It was possible a reason that root repair can restore knee biomechanics closed to normal levels, which has been reported by biomechanical studies.^15^

Our findings are similar to previous results^16^,^17^ that the pathological changes in the meniscus and how to manage them are associated with PFJ osteoarthritis. In addition, pathological changes in the lateral meniscus had a stronger effect on the lateral aspect of the patellofemoral joint than the medial meniscus did on the medial patellofemoral joint. A biomechanical study by Bai et al^18^ found that meniscectomies at different sites resulted in increased pressure or abnormal pressure distribution in the patellofemoral joint, leading to postoperative degenerative patellofemoral arthritis. Similarly, Keays et al^19^ considered meniscectomy as a predictor in patellofemoral arthritis in patients after anterior cruciate ligament reconstruction in a prospective study. However, unlike previous studies, the present study focused firstly on the effect of the posterior root of the lateral meniscus after ACL reconstruction and secondly on the tearing or suturing of the posterior root of the lateral meniscus rather than meniscectomy.

Thus far, different findings also exist that have been reported by several researchers. In a study addressing the analysis of predictors for patellofemoral pain after anterior cruciate ligament reconstruction. Culvenor et al^20^ reported that older age at the time of surgery was the only predictor of postoperative prepatellar pain, although pre-operative patellofemoral pain level, activity level, patellofemoral cartilage damage, and meniscectomy were evaluated discriminant analysis. Furthermore, another meta-analysis showed that in patients with ACL deficiency, medial meniscal tear or meniscectomy increased the risk of knee osteoarthritis, whereas lateral meniscal injury or meniscectomy was not associated with the development of knee osteoarthritis.^21^ We consider that the measurement of patellofemoral joint pressure values and the study of postoperative patellofemoral conditions rather than total knee conditions may account for the difference between these results and those of the present study.

### Limitations:

First, as an immediate cadaveric study, the study ignored the effect of the dynamic structure and healing of the joint on the outcome. Second the loading values applied to the quadriceps muscles were constant. Third, although the trial had shown differences in patellofemoral joint pressure data, the clinical implications of such differences remain to be seen.

## CONCLUSIONS

This paper investigates the effect of posterior root tear and repair of the lateral meniscus on patellofemoral contact stress of the knee after ACL reconstruction. The results showed that the posterior root structure of the lateral meniscus could exert pressure on the patellofemoral joint in the knee with ACL reconstruction at 30° of flexion. These results of our study may provide information for counseling patients on the choice of treatment for the posterior root of the lateral meniscus after ACL reconstruction.

### Authors’ Contributions:

**QZ** and **SG:** Designed this study and prepared this manuscript, and are responsible and accountable for the accuracy or integrity of the work.

**JD** and **XZ:** Collected and analyzed clinical data.

**KK:** Data analysis, **s**ignificantly revised this manuscript.
